# *Bonellia albiflora*: A Mayan Medicinal Plant That Induces Apoptosis in Cancer Cells

**DOI:** 10.1155/2013/823453

**Published:** 2013-06-17

**Authors:** Rosa Moo-Puc, Juan Chale-Dzul, Edgar Caamal-Fuentes

**Affiliations:** Unidad de Investigación Médica Yucatán, Unidad Médica de Alta Especialidad, Centro Médico Ignacio García Téllez, Instituto Mexicano del Seguro Social, 41 No. 439 x 32 y 34, Colonia Industrial CP, 97150 Mérida, YUC, Mexico

## Abstract

Few studies have been carried out on the medical flora of Mexico's Yucatan Peninsula in search for new therapeutic agents, in particular against cancer. In this paper, we evaluated the cytotoxic potential of the extract of *Bonellia albiflora*, a plant utilized in the traditional Mayan medicine for treatment of chronic injuries of the mouth. We carried out the methanolic extracts of different parts of the plant by means of extraction with the Soxhlet equipment. We conducted liquid-liquid fractions on each extract with solvents of increasing polarity. All extracts and fractions were evaluated for cytotoxic activity versus four human cancer cell lines and one normal cell line through a tetrazolium dye reduction (MTT) assay in 96-well cell culture plates. The methanolic root-bark extract possessed much greater cytotoxic activity in the human oropharyngeal cancer cell line (KB); its hexanic fraction concentrated the active metabolites and induced apoptosis with the activation of caspases 3 and 8. The results demonstrate the cytotoxic potential of the *B. albiflora* hexanic fraction and substantiate the importance of the study of the traditional Mayan medicinal plants.

## 1. Introduction

Traditional medicine is a practice that has been carried out from antiquity to our present time by inhabitants of the indigenous pueblos of Mexico, among which the Mayan population of the Yucatan Peninsula in Mexico is included. In the traditional Mayan medicine, plants are of great importance, which can be considered as evidence of their effectiveness for the control of many types of diseases. Likewise, they comprise one of the most important alternatives for health care, above all, in communities where primary health services are not accessible. In addition, they can be taken advantage of widely as a natural renewable resource. Together with what was previously described, the traditional medicine of the indigenous pueblos was recognized by the World Health Organization (WHO), which caused a powerful drive toward the research of medicinal plants [1]. 

The Mayan ethnobotanical literature in its majority is composed of historic or descriptive studies, in whose contents there predominates a compendium of diseases and treatments known to Mayan healers of distinct eras [2, 3]. The Mayan people knew of and treated distinct diseases, including those of infectious origin (intestinal infections, infectious dermatitis, and respiratory infections), chronic diseases (asthma, fatigue, nephritis, and hypertension), and psychological-type diseases (insomnia, nervousness, and hysteria). In addition, they cured other illnesses such as the following: abscesses; calluses; corns, hard protuberances; polyps; tumors; and warts or sores, generally tangible or visible on the skin [1, 4].

In the traditional Mayan medicine of the Yucatan Peninsula, “cancer” is known as an illness or a set of illnesses that can manifest themselves as an affectation of the skin or subadjacent muscle mass, or an affectation in the form of pain in some internal organ. The term alludes to a difficult-to-cure illness or a one with a disagreeable aspect (when it affects the skin); if it is an internal cancer, the patient's semblance reveals the disease. The old inhabitants assigned names in the Mayan language to this set of symptoms; in the Mayan tongue, “cancer” is known as “*tsunuz”* or “*tsunuztacan”*, and hard protuberances or tumors are known as “*chu'uchum”* [3, 5].

Prior studies have demonstrated that the extracts of plants utilized in the traditional Mayan medicine for the treatment of the signs and symptoms suggestive of cancer possess cytotoxic activity [6]. Similarly, two studies conducted on two species of the genus *Bonellia *(*Bonellia macrocarpa* and *Bonellia flammea*) from the Yucatan Peninsula reveal the presence of novel compounds, such as active agents with anticarcinogenic activity [7, 8]. Within this context, the Yucatan Peninsula has five species of the genus *Bonellia*, among which the species *B. macrocarpa*, *B. flammea*, and *B. albiflora* are employed in the traditional Mayan medicine for the treatment of the dermatological-type afflictions [5, 9, 10]. Of these three species, only *B. albiflora* has not been the object of any phytochemical or biological activity study. *B. albiflora* is denominated “Si'ik” in the traditional Mayan medicine and is used as an antitussive for the treatment of skin and mouth wounds and to relieve toothache pain [10]. In this work, we proposed an evaluation of the cytotoxic potential of the organic extracts of *B. albiflora*.

## 2. Materials and Methods

### 2.1. Plant Material

*Bonellia albiflora* (Lundell) B. Ståhl and Källersjö was collected from different Localities of the State of Yucatan, Mexico, during the summer of 2010. Plant material was identified and authenticated by taxonomists from the Department of Natural Resources of the Scientific Research Center of Yucatan (CICY).

### 2.2. Chemicals

Dulbecco's modified Eagle's medium (DMEM), heat-inactivated fetal bovine serum (FBS), and penicillin and streptomycin (PS) were purchased from Gibco, Carlsbad, CA, USA. The 3-(4-5-dimethylthiazol-2-yl)-2,5-diphenyl tetrazolium bromide (MTT), dimethyl sulfoxide (DMSO), and etoposide were purchased from Sigma, St. Louis, MO, USA. Caspase assay kits and apoptotic DNA laddering kit were purchased from BioVision Research Products, Palo Alto, CA, USA.

### 2.3. Extraction and Fractionation

Each vegetal part was separated, dried, and pulverized. Dried powder of the separated plant material (100 g) was exhaustively extracted using a Soxhlet apparatus at 60°C of temperature with methanol (500 mL). The supernatants were filtered and evaporated under vacuum by means of a rotaevaporator to obtain a dried extract. The methanol extract of each vegetal material (10 mg) was suspended in 20 mL methanol : water (1 : 3) and extracted successively using 50 mL of solvents of increasing polarity: hexane, dichloromethane, and ethyl acetate, such that the final residue extract was an aqueous fraction. The fingerprint of active hexane extract (5 mg) was obtained for gas chromatography-mass spectrometry (GC-MS).

### 2.4. Cell Lines and Culture

Cell lines of the oropharyngeal carcinoma (KB ATCC-CCL-17), laryngeal carcinoma (Hep-2), cervix adenocarcinoma (HeLa ATCC-CCL-2), and cervix squamous carcinoma (SiHa ATCC-CCL-35) as well as one normal cell line, canine cell kidney (MDCK ATCC-CCL-34), from the American Type Culture Collection (ATCC) were kindly provided by Veronica Vallejo-Ruíz from the East Biomedical Research Center-IMSS. The cells were cultured in DMEM medium, containing 10% SFB supplemented with 100 units/mL penicillin G and 100 *μ*g/mL streptomycin in 5% CO_2_-95% humidified air at 37°C.

### 2.5. Cytotoxicity Assay

The cytotoxicity was determined by the MTT assay according to the method described by Denizot and Lang [11] with some modifications. Briefly, 5 × 10^3^ viable cells from each cell line were seeded in a 96-well plate and incubated for 24–48 h. When cells reached >70% confluence, the medium was replaced and the cells were treated with the extract dissolved in DMSO (maximum concentration of 0.05%) at 2.34 to 300 g/mL. After 48 h of incubation, 10 *μ*L MTT (5 mg/mL) was added to each well and incubated at 37°C for 4 h. The medium was removed, and the formazan precipitate was dissolved in 100 *μ*L of acidified isopropanol (0.4 N HCl). The optical density was determined with a spectrophotometer at 540 nm. Cells treated with 0.05% DMSO and docetaxel were used as negative and positive controls, respectively. The concentration of the extract that killed 50% of the cells (CC_50_) was calculated by GraphPad Prism 4.00 software. All determinations were performed in triplicate. MDCK cell line was used to evaluate the selective index (SI) of extracts. SI is defined as the ratio of cytotoxic activity from normal cell and cancer cell lines.

### 2.6. GC-MS Analysis

The chromatographic separation was carried out by GC-MS analysis on an Agilent gas chromatograph, model 6890N, coupled to a mass selective detector, model 5975B. Compounds were separated on a DB-5 ms capillary column (30 m × 0.32 mm i.d., 0.25 *μ*m film thickness) (J&W Scientific, Folsom, CA, USA). One microliter of the sample was injected into GC-MS using split mode (50 : 1). The injector temperature was 250°C. The column temperature was programmed as follows: initial temperature at 160°C for 3 min, 10°C/min to 240°C, 240°C for 2 min, 5°C/min to 250°C, 250°C for 10 min, 5°C/min to 300°C, and 300°C for 10 min. Mass detector conditions were the following: electronic impact (EI) mode at 70 eV; source temperature: 230°C; scanning rate: 1 scan/s; mass acquisition range: 20–600 amu; solvent delay, 4 min. Carrier gas was helium at 1 mL/min. Volatile components were tentatively identified by comparing their mass spectra using NIST Standard Reference Database Version NIST 05 for Windows. An authentic standard of bonediol compound was kindly provided by Dr. Peraza-Sánchez from CICY.

### 2.7. Analysis of DNA Fragmentation

DNA fragmentation was determined according to the method described by Tong et al. [12]. Briefly, the cells were treated with the extract at 10 and 50 *μ*g/mL and incubated for 6, 12, and 24 h. After incubation, the cells were harvested by centrifugation and washed twice in ice-cold PBS. An apoptotic DNA laddering kit (BioVision apoptotic DNA ladder extraction kit) was used to isolate DNA according to the manufacturer's protocol; the DNA in the samples was separated on 1.5% agarose gel containing 1 *μ*g/mL of ethidium bromide. DNA bands were visualized under ultraviolet illumination and were photographed.

### 2.8. Assays of Caspases Activities

Caspases 3, 8, and 9 activities were performed using FLICE/Caspase Colorimetric assay kit, following the manufacturer's protocols. Briefly, 5 × 10^6^ cells treated with 10 or 50 *μ*g/mL extract for 6, 12, or 24 h were harvested, washed with PBS, and centrifuged at 800 ×g for 10 min at 4°C. The cell pellets were resuspended in 50 *μ*L lysis buffer and incubated on ice for 10 min before being centrifuged at 10,000 ×g for 1 min. The supernatant was collected in a 1.5 mL tube and kept on ice. After measuring protein concentration, 200 *μ*g of protein was dissolved in 50 *μ*L cell lysis buffer. The reaction buffer with 10 mM DDT was added to each sample. Finally, a specific substrate for each caspase (DEVD-*ρ*NA, IETD-*ρ*NA, and LEHD-*ρ*NA) was added to the samples, incubated at 37°C for 1 h, and read at 405 nm. The enzyme activity was expressed as fold over control sample.

## 3. Results and Discussion

### 3.1. Cytotoxic Activity of Methanolic Extracts

The cytotoxicity results of the methanolic extracts from different parts of *B. albiflora *are summarized in Table 1. The root bark's methanolic extract exhibited the most interesting cytotoxic activity compared to extracts of *B. albiflora* leaves and stem bark, with a CC_50_ of 12–31 *μ*g/mL on the four human cancer cell lines. KB cell line showed a greater sensitivity to the extract with a CC_50_ of 12.64 *μ*g/mL. The nontumor canine kidney cell line MDCK was less sensitive to the effects of the extract with an SI of >5 in the cell lines evaluated (Table 1). The US National Cancer Institute (NCI) has proposed that crude extracts with potential cytotoxic activity are those presenting a CC_50_ of ≤30 *μ*g/mL; thus, this extract was identified as important for future studies [13]. These data are similar to those obtained in active *B. macrocarpa*-root methanolic extracts on human cell lines: KB, prostate adenocarcinoma (PC3), cervix squamous carcinoma (SiHa), breast adenocarcinoma (MCF-7), cervix adenocarcinoma (HeLa), and laryngeal carcinoma (Hep-2) [6].

The extract of leaves was the second in greatest activity, only on KB cell line with a CC_50_ of 23.85 *μ*g/mL according to NCI criteria, followed by that of the stem bark's extract, which was less cytotoxic to KB and Hep-2 cell lines.

### 3.2. Cytotoxic Activity of Fractions

The methanolic extracts of different parts of the plant were fractionated with solvents of increasing polarity for later cytotoxicity studies in the cell lines. The hexanic fraction obtained from the liquid-liquid partitioning of the methanolic extract of root bark (HFBa) presented superior cytotoxic effects compared to the original extract, with a CC_50_ between 2 and 27 *μ*g/mL in the distinct cell lines (Table 2). The hexanic fraction's SI also improved compared to the original extract in the cell lines evaluated (SI = 5–54). The methanolic fractions of the bark and leaves extracts were not active at concentrations of  >200 *μ*g/mL (data not shown).

Previously, we conducted a bioguided study to evaluate the antiproliferative activity of *B. macrocarpa, *yielding the isolation of the compound bonediol, which showed moderate activity in cancer cell lines [8]. However, the present study did not show cytotoxic effects with HFBa comparable to the original methanolic extract in the cell lines evaluated (Table 2). An explanation to these results may be that bonediol inhibits some point of cellular proliferation (cycle cell or replication of DNA), while the effects that are observed in the cytotoxic assay are damage or general toxicity (apoptosis or necrosis) [14].

HFBa presented better cytotoxic effects compared to bonediol and was more selective toward the tumor than toward normal cells; SI is considered an indicator of biological activity and is not related to cytotoxicity if the SI is >10 [15]. In this regard, only HFBa satisfied these criteria and was more potent in the KB cell line with a CC_50_ of 2.73 *μ*g/mL; this cell line is related to oral cancer and is in agreement with the plant's use in the traditional Mayan medicine for chronic oral lesions [10], a term that could be related with cancer.

### 3.3. GC-MS Analysis

**Figure 1:**
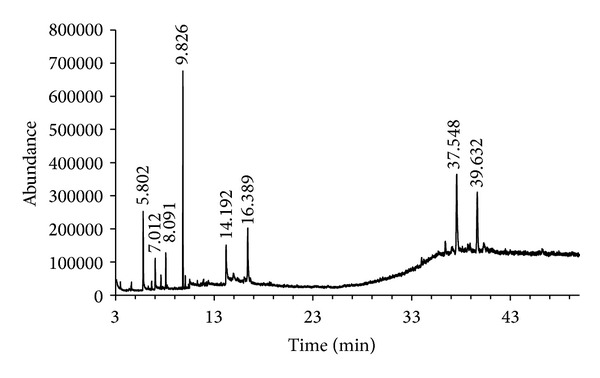
Gas chromatography of HFBa.

Identification and chemical analysis of bioactive hexane fraction by GC-MS is displayed in Table 3. The chromatogram revealed a total of eight peaks, six of which were identified by the database: dodecanoic acid; tridecanoic acid; 2-nonyl-malonic acid, dimethyl ester; stigmasta-7,16-dien-3-ol; 9,19-Cyclo-lanost-24-en-3-ol; and bonediol. This last one was identified by retention time and comparison of the mass spectrum of an authentic standard previously isolated from *B. macrocarpa* [8]. The major components found were the following: 2-nonyl-malonic acid, dimethyl ester (37.39%), followed by stigmasta-7,16-dien-3-ol (13.63%), dodecanoic acid (13.22%), 9,19-Cyclo-lanost-24-en-3-ol (9.90%), and bonediol (8.98%). Unidentified components with retention times of 8.092 (6.22%) and 14.207 (5.38%), as well as n-tridecanoic acid (5.25%), were minor compounds in the HFBa (Figure 1).

Bonediol was isolated from the methanolic extract of *B. macrocarpa *roots as a bioactive component. In this work, we detected the presence of this compound at a low concentration; thus, it could be referred to as a possible chemotaxonomic marker. Additionally, in other species such as *B. pungens*, a triterpene has been isolated [16], and from *B. ruscifolia*, two triterpenes have been isolated, without reports of biological activity [17, 18]. In this work, we have only found evidence of the presence of a lanosterol-derived triterpene in the active hexanic fraction. In addition, we detected a ubiquitinated sterol, a derivative of stigmasterol. To our knowledge, this is the first time that both compounds have been reported in this genus.

In recent years, not only has the study of medically bioactive compounds from plants become more frequent, but also that of the plant extracts themselves or the mixture of compounds that together could yield better biological activity than that exhibited by a single compound has, moreover, become frequent [19]. In this work, the components are described as the fingerprinting of HFBa performed by GC-MS for future standardizations.

### 3.4. DNA Fragmentation

**Figure 2:**
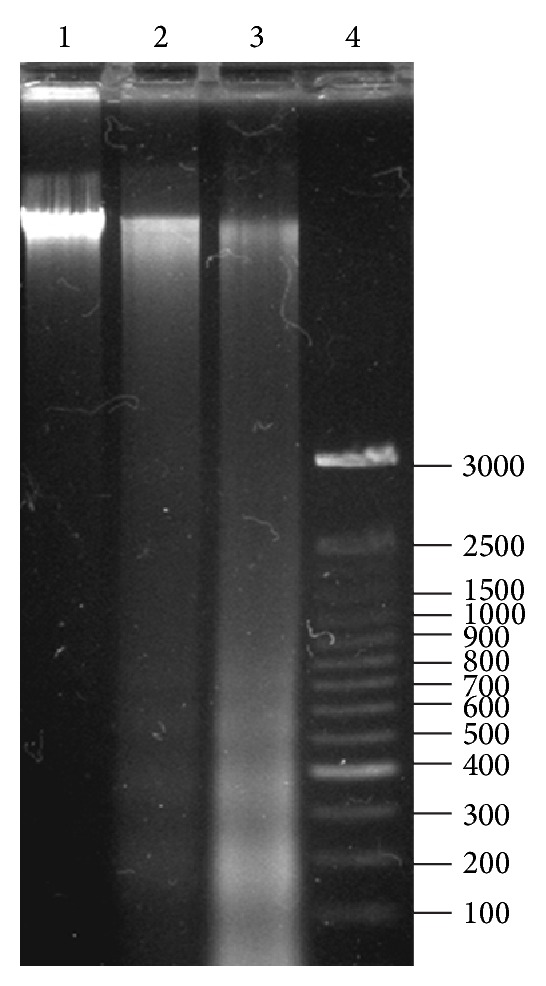
Effect of hexane root extract *Bonellia macrocarpa* on DNA fragmentation in KB cells. After the treatment of the cells with a concentration of 50 *μ*g/mL of *B. macrocarpa *for 12 h, DNA was isolated and separated on 1.5% agarose gel. DNA was stained with ethidium bromide and visualized under UV light. Lanes 1 to 4: lane 1 (negative control): DNA collected from untreated KB cells after 18 h; lane 2 (positive control): DNA collected from KB cells treated with 50 *μ*g/mL of etoposide after 18 h; lane 3: DNA collected from KB cells treated with 50 *μ*g/mL of extract after 18 h; lane 4: DNA molecular weight marker.

We observed that the methanol extract of the roots of *B. albiflora* showed typical morphology of apoptosis (data not shown) on KB cell lines. Similarly, HFBa was shown to induce apoptotic morphology on KB cell lines. These results led us to evaluate whether the hexanic fraction that demonstrated the greatest cytotoxicity and apoptosis morphological characteristics in the KB cell line could induce this process; thus, we evaluated the fragmentation of DNA, typical of the process of apoptosis. DNA fragmentation was registered from lesser to greater magnitude within a treatment concentration range of 10 or 50 *μ*g/mL and an incubation-time range of 6–24 h.  Figure 2 shows typical DNA fragmentation in KB cells after treatment with 50 *μ*g/mL HFBa and an 18 h incubation period. Several studies have shown the apoptotic effect of certain plant methanolic extracts [20–24]. However, few studies have investigated the chemical characteristics of the compounds that may possess this activity. In those few studies, it was generally found that the low-polarity organic fractions are responsible for the apoptotic effect on the cell lines, coinciding with the results obtained in this study [25, 26].

It is unknown whether the compounds in HFBa are responsible for apoptosis induction, but it cannot be attributed to a single compound such as bonediol that, although present in the extract, requires high concentrations to induce apoptosis (data not shown), unlike HFBa, which induces DNA fragmentation at 10 *μ*g/mL. With regard to the above, in addition to bonediol, we report the presence of dodecanoic acid and a derivative of stigmasterol as components of HFBa. These compounds have been associated with cytotoxic activity observed for the hexane fraction of *Crocus sativus* [27]. Furthermore, some authors have demonstrated that derivatives of stigmasterol showed significant cytotoxic activity in cancer cell lines that depended on apoptosis [28–31]. In particular, spinasterol (stigmasta-7, 22-dien-3beta-ol) has demonstrated decreased incidence of skin tumors *in vivo *[32]. In fact, spinasterol and the derivative reported in this study differ in double enlace at position 22 in spinasterol and position 16 for the stigmasterol derivative. Perhaps, stigmasta-7, 22-dien-3beta-ol could contribute to cytotoxic activity observed in this study. In addition, it is known that lanostanes are a group of tetracyclic triterpenoids derived from lanosterol, which have multiple activities against cancer cells including the induction of apoptosis [33, 34]. Possibly, the lanostane- and stigmasterol-type compounds reported in the active hexane fraction have a degree of cytotoxic activity and an effect of inducing apoptosis. It is likely that several compounds present in the hexane fraction act synergistically to induce cytotoxicity and apoptosis.

### 3.5. Analysis of the Activity of Caspases

**Figure 3:**
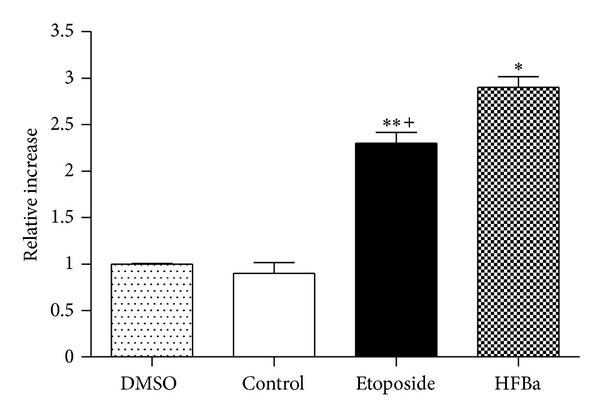
Treatment during six hours with *Bonellia macrocarpa* hexane fraction induced caspase 8 activation. Treatments were the following: DMSO (0.05%), control (no treatment), etoposide (50 *μ*g/mL), and HFBa (50 *μ*g/mL). Each symbol is the mean ± SD relative caspase activation from three assays, normalized with the control group. One-way ANOVA: *F*(3, 11) = 96.84, *P* < 0.0001; Tukey's post-hoc test: *P < 0.001 versus DMSO and control group; **P < 0.001 versus DMSO and control group; ^+^ *P* < 0.05 versus HFBa.

**Figure 4:**
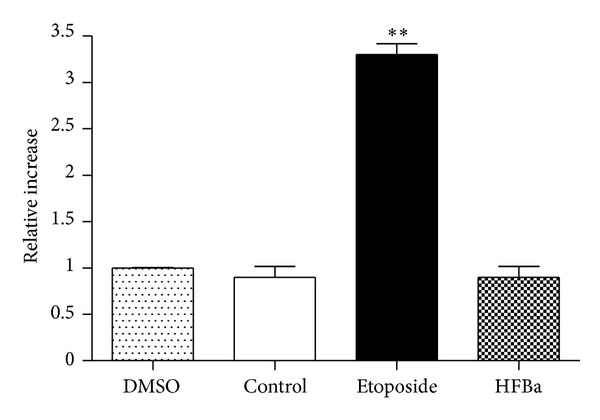
Treatment during 12 hours with *Bonellia macrocarpa* hexane fraction did not induce caspase 9 activation. Treatments were the following: DMSO (0.05%), control (no treatment), etoposide (50 *μ*g/mL), and HFBa (50 *μ*g/mL). Each symbol is the mean ± SD relative caspase activation from three assays, normalized with the control group. One-way ANOVA: *F*(3, 11) = 140.11, *P* < 0.0001; Tukey's post-hoc test: **P <  0.001 versus all groups.

**Figure 5:**
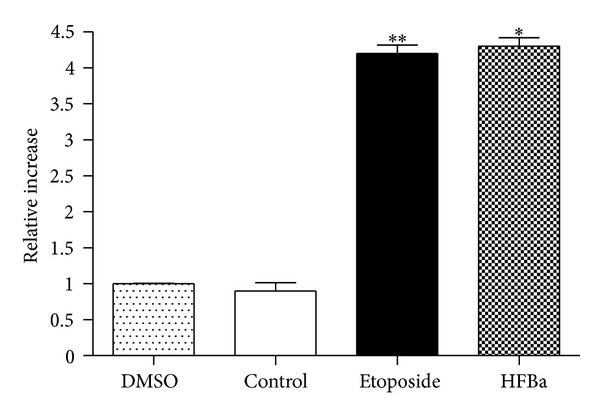
Treatment during 12 hours with *Bonellia macrocarpa* hexane fraction induced caspase 3 activation. Treatments were the following: DMSO (0.05%), control (no treatment), etoposide (50 *μ*g/mL), and HFBa (50 *μ*g/mL). Each symbol is the mean ± SD relative caspase activation from three assays, normalized with the control group. One-way ANOVA: *F*(3, 11) = 363.2, *P* < 0.0001; Tukey's post-hoc test: **P < 0.001 versus DMSO and control group; *P < 0.001 versus DMSO and control group.

To know whether the mechanism of activation of DNA fragmentation was induced by activation of apoptosis via the intrinsic or extrinsic pathway, we evaluated the activity of caspases that is characteristic of each. The incubation periods were of 2, 4, 6, and 12 h to obtain an activation profile. Caspase 8 was activated after 6 h of treatment with 50 *μ*g/mL of HFBa; the increase was three times greater compared to control cells without treatment (negative control) (Figure 3). No increase was observed in the activation of caspase 8 in the 2, 4, and 12 h incubation periods. Caspase 9 was not activated in KB cells after treatment with 50 *μ*g/mL of HFBa during 2–12 h, which suggests a lack of apoptosis activation by the intrinsic pathway (Figure 4). Caspase 3 activity increased four folds compared to that of the control in HFBa-treated cells, which is in agreement with caspase 8 activation (Figure 5). The increase of caspase 8 activity is typical of extrinsic-pathway activation of apoptosis, which in turn activates other procaspases, among these is caspase 3, which in turn leads to the degradation of nuclear proteins such as laminin A, fodrin, actin, and gelsolin. It also leads to the release of the caspase-activated DNase protein inhibitor (ICAD), converting it into the caspase-activated DNase (CAD) enzyme, whose objective is DNA degradation [35], as depicted in Figure 2. Given that caspase 9 was not activated, we conclude that *B. albiflora *induces apoptosis by the extrinsic pathway. This evidences the great potential that this fraction possesses as an alternative or adjunct therapy in the treatment of cancer. In the literature are several studies of extracts from plants and their effects on the induction of the intrinsic apoptosis pathway [36–42]. However, few studies have shown the activation of the extrinsic apoptosis pathway by plant extracts; for example, the methanolic extract of *Paeonia suffruticosa *induces apoptosis in the human stomach cancer cell line (AG3) [43], and dandelion root extract has the capacity to induce apoptosis in the chronic myelomonocytic leukemia cell line (CMML) and in drug-resistant melanoma cells [44, 45]. It is not known which of the compounds present in the active fraction of *B. albiflora* may cause activation of the extrinsic pathway of apoptosis. Regardlessly, there are reports of the induction of apoptosis by activation of extrinsic signaling mediated by natural and synthetic triterpenoids [46–48]. Additionally, the lanostane-type triterpenoid, polyporenic acid C isolated from *Poriacocos*, induces caspase-8-mediated apoptosis in human lung cancer [49]. Notably, *β*-sitosterol (structural isomer of stigmasterol) has been shown to induce apoptosis by activation of the extrinsic pathway, activating Fas signaling in human breast cancer cells [50]. This might suggest that both the triterpene and sterol components of the extract used in this study would have a major role in the induction of apoptosis mediated by the activation of the extrinsic pathway. 

To our knowledge, this is the first time it has been demonstrated that the extract of a plant employed in the traditional Mayan medicine possesses an effect on apoptosis. Future studies will be directed toward standardizing the extract and evaluating the latter with *in vivo* models. Additional studies are necessary for elucidating the compounds responsible for the observed cytotoxic activity and their exact mechanism of apoptosis.

## 4. Conclusions

The hexanic fraction of *B. albiflora* roots exerts cytotoxic effects and induces apoptosis via the extrinsic pathway, which suggests its potential for the treatment of cancer. We suggest the complete isolation of the components present in the hexane fraction of *B. albiflora* for evaluation in the cytotoxic assay and induction of apoptosis, to elucidate which are the active compounds as well as to understand the mechanism of action.

## Figures and Tables

**Table 1:** Cytotoxicity (CC_50_) of methanolic extracts from *B. albiflora*.

Extract	Cell lines CC_50_ *μ*g/mL (selective index)				
	MDCK	KB	HeLa	Hep-2	SiHa
Leaves	91.39	23.85 (3.83)	47.05 (1.94)	35.20 (2.59)	47.45 (1.92)
Stem bark	249.40	62.30 (4.00)	NA	72.30 (3.45)	NA
Root bark	173.52	12.64 (13.72)	31.85 (5.44)	35.34 (4.91)	31.50 (5.50)
Docetaxel	1.10	0.23 (4.78)	0.20 (5.50)	0.08 (13.75)	0.32 (3.43)

NA: no activity > 200 *μ*g/mL.

**Table 2:** Cytotoxicity of organic fractions from methanolic extract of *B. albiflora* root bark and bonediol.

Extract	Cell lines CC_50_ *μ*g/mL (selective index)				
	MDCK	KB	HeLa	Hep-2	SiHa
Hexane	148.48	2.73 (54.38)	14.29 (10.39)	15.48 (9.59)	27.02 (5.49)
Dichloromethane	NA	NA	NA	NA	NA
Ethyl acetate	NA	NA	NA	NA	NA
Aqueous	NA	NA	NA	NA	NA
Bonediol	139.71	80.60 (1.73)	115.45 (1.21)	92.50 (1.51)	54.40 (2.56)
Docetaxel	1.10	0.23 (4.78)	0.20 (5.50)	0.08 (13.75)	0.32 (3.43)

NA: no activity > 200 *μ*g/mL.

**Table 3:** Chemical composition of hexane fraction of *B. albiflora*.

Peak no.	Retention time (min)	Peak relative (%)	*m*/*z* (relative abundance %)	Component
1	5.802	13.221	200 (10), 171 (10), 157 (30), 143 (10), 129 (40), 115 (20), 101 (15), 85 (30), 73 (100), 60 (85), 43 (70), 29 (40).	Dodecanoic acid
2	7.012	5.256	214 (10), 185 (10), 171 (35), 157 (5), 143 (5), 129 (40), 115 (25), 97 (15), 85 (20), 73 (95), 60 (85), 43 (70), 29 (35).	Tridecanoic acid
3	8.091	6.222	208 (19), 166 (13), 152 (100), 137 (18), 121 (6), 107 (5), 91 (13), 77 (13), 55 (5), 41 (13), 28 (19).	Unidentified
4	9.826	37.396	259 (5), 156 (7), 145 (70), 132 (100), 113 (13), 100 (20), 87 (18), 69 (16), 55 (33), 41 (31), 29 (13).	2-Nonyl-malonic acid, dimethyl ester
5	14.1927	5.381	350 (100), 209 (80), 195 (24), 179 (48), 164 (12), 151 (16), 136 (2), 75 (8), 57 (8), 43 (20), 28 (26).	Unidentified
6	16.389	8.983	294 (70), 209 (13), 179 (10), 153 (100), 139 (5), 123 (20), 77 (9), 41 (20).	Bonediol
7	37.548	13.634	412 (22), 369 (10), 341 (10), 300 (15), 271 (80), 246 (20), 207 (90), 173 (10), 147 (40), 107 (43), 81 (75), 55 (80), 43 (100), 28 (40).	Stigmasta-7,16-dien-3-ol
8	39.632	9.907	426 (25), 411 (100), 393 (45), 259 (10), 215 (10), 187 (15), 173 (15), 161 (15), 135 (25), 109 (40), 69 (90), 55 (40), 41 (45).	9,19-Cyclo-lanost-24-en-3-ol
